# A survey of digital radiography practice in four South African teaching hospitals: an illuminative study

**DOI:** 10.2349/biij.6.1.e5

**Published:** 2010-01-01

**Authors:** T Nyathi, TF Chirwa, DG van der Merwe

**Affiliations:** 1 School of Physics, University of the Witwatersrand, Johannesburg, South Africa; 2 Epidemiology and Biostatistics Unit, School of Public Health, University of the Witwatersrand, Johannesburg, South Africa; 3 Charlotte Maxeke Johannesburg Academic Hospital, Johannesburg, South Africa

**Keywords:** Digital radiography, radiography practice, quality control

## Abstract

**Purpose:**

The purpose of this study was to assess radiographer familiarity and preferences with digital radiography in four teaching hospitals and thereafter make recommendations in line with the migration from screen film to digital radiography.

**Materials and methods:**

A questionnaire was designed to collect data from either qualified or student radiographers from four teaching hospitals. From the four teaching hospitals, there were a total of 205 potential respondents. Among other things, responses regarding experiences and preferences with digital radiography, quality control procedures, patient dose, advantages and disadvantages of digital radiography were sought. The information collected was based on self-reporting by the participants. The study is exploratory in nature and descriptive statistics were generated from the collected data using Microsoft Excel 2007 and StatsDirect software.

**Results:**

Sixty-three out of 205 (31%) radiographers from all the four radiology centers responded to the circulated questionnaire. Only 15% (8) of the qualified radiographers had 4 or more years of experience with digital radiography compared to 68% (36) for the same amount of experience with screen-film radiography. Sixty-one percent (38) of the participants had been exposed to digital radiography during their lectures while at university. A small proportion, 16% (10) of the respondents underwent formal training in quality control procedures on the digital X-ray units they were using. Slightly more than half (55%) of the participants felt it was easier for them to retake an image in digital radiography than in screen film radiography.

**Conclusion:**

The results of this survey showed that the participants are familiar with digital radiography and have embraced this relatively new technology as shown by the fact that they can identify both its advantages and disadvantages as applied to clinical practice. However, there are minimal quality control procedures specific to digital radiography being undertaken as such there is need for formal education, continuing education and manufacturer training with respect to quality control as institutions make the transition from conventional screen film radiology to digital radiology.

## INTRODUCTION

The foundation of diagnostic radiology lies in the discovery of X-rays by Professor Wilhelm Conrad Röentgen, of the University of Wurzburg, Germany, in November 1895 [1]. Radiography has evolved over the years from using screen-film technology to digital imaging, which is sometimes referred to as filmless radiography. Nowadays, digital X-ray units are ubiquitous in most radiology departments [2].

Digital imaging is a term used to describe general radiography when the radiographic images are in digital form and are capable of being displayed on a computer monitor [3]. Digital imaging can be realized through the use of either computed radiography or digital radiography. It has become technically possible and economically feasible for digital imaging technologies to challenge screen-film technology for projection radiography [4]. This has been made possible by the prerequisite technological advances such as high-luminance and high-resolution display monitors combined with high-performance computer workstations and a decline in the price of computer technology. This shift in choice of imaging modality is not only confined to developed countries but is gradually finding its way to developing countries.

Computed radiography uses an imaging plate coated with photostimulable phosphors to capture x-rays as they traverse through the patient. BaFBr:Eu^2+^ and BaFI:Eu^2+^ are the commonly used phosphors [5]. When exposed to radiation, the phosphors absorb and store x-ray energy in gaps of their altered crystal structure. This trapped energy comprises a latent image. When stimulated by additional light energy of the proper wavelength, the trapped energy is released. The amount of light emitted is directly proportional to the number of X-ray photons absorbed. The resulting computed radiography image comprises of multiple rows and columns of pixels representing the X-ray intensities at locations (x;y). Eventually, these raw pixel values are processed using mathematical algorithms for subsequent display.

In digital radiography, the digitization of the X-ray projection image occurs within the image receptor. Detectors in digital radiography can be in the form of charged coupled devices or flat panel imagers. Furthermore, flat panel imagers are generally of two types namely, direct conversion or indirect conversion systems. Direct conversion systems use an X-ray sensitive photoconductor layer (amorphous selenium, a-Se) and a thin-film transistor (TFT) charge collector [5, 6, 7]. Radiation absorbed by the photoconductor is directly converted into charge, which is drawn to the TFT charge collector where it is stored until readout. On the other hand, indirect conversion systems use scintillators e.g. cesium iodide (CsI) or gadolinium oxysulphide (Gd_2_O_2_S) layered on top of an array of light-sensitive photodiodes with TFTs [5, 6, 7]. The scintillator converts radiation into light that is detected by the photodiode/TFT array. For anatomical regions with gross density differences such as the chest, thoracic spine, shoulder, facial bone, cervical spine, thoraco-lumbar spine, femur and feet, digital radiography has shown superior image quality over screen-film radiography [3].

In this work, the term digital radiography will represent both computed radiography and digital radiography. Digital radiography has both advantages and disadvantages when compared to screen-film radiography, as summarized in Table 1 based on literature [8, 9, 10].

**Table 1:** Advantages and disadvantages of digital radiography over screen-film radiography.

Advantages	Disadvantages
• Increased dynamic range • Linear response of images • Availability of post-processing functions • Easy to archive since images are in digital format • Leads to a higher patient thorough-put • Separation of image capture, processing, storage and display processes which means they can be optimized individually	• Poorer spatial resolution. • Artifacts due to the imaging plate, image processing algorithms etc • Non-availability of post-processing functions • Increased sensitivity to scattered radiation. • More expensive than screen-film radiography. • Lack of familiarity to radiologists and radiographers.

Over the last few years, public hospitals in South Africa have been purchasing digital units for their radiology departments. This is in line with worldwide trends of migration from screen-film radiography to digital radiography. Upon adoption of new technology, it is advisable that the technology undergoes evaluation and critique so that strategies are devised to optimize its use. Currently, there is no published data on use of digital X-ray units in South Africa except for one paper on computed radiography in mammography [11]. The transition from screen-film to digital radiography technology is not a simple matter, at it involves acquirement of new skills, change in the workflow process, training and retraining at times [7]. As such, this present study seeks to elucidate two issues, which are as listed below:

- Assess radiographers’ familiarity and preferences with digital radiography.
- Make recommendations in line with migration from screen film radiography to digital radiography to the participating institutions.

## MATERIALS AND METHODS

This was a cross-sectional study in which a questionnaire was designed to collect data from either qualified or student radiographers from four teaching university hospitals in South Africa. Only qualified radiographers or registered student (trainee) radiographers were eligible for inclusion in the current study. Student radiographers were included in the study because they would give an insight into the training programs and in some cases due to staff shortages they work under minimal supervision. From the four teaching hospitals, there were a total of 205 potential respondents. Due to a request by one of the participating institutions, the hospitals will not be identified by name in this study.

The questionnaire used took a multiple format, i.e. it had closed- and open-ended questions. The information collected was based on self-reporting by the study participants. The questionnaire captured the participants’ familiarity, preferences, knowledge and workmanship with regards to digital radiography. The participants in this survey were further asked questions relating to operation of their digital X-ray units, comparing digital radiography to screen-film radiography and their preferences when using digital radiography units. The questionnaire captured the quality control procedures performed at the different institutions and, furthermore, the participants were asked to identify the advantages and disadvantages of digital radiography.

A soft copy of the questionnaire was e-mailed to the radiotherapy medical physicist at the relevant teaching hospital, who made printouts and hand delivered them to the Assistant Director of Radiography. The Assistant Director then asked the radiographers to respond to the questionnaire. Participants were given a maximum of one week to respond to the questionnaire. Participation in the study was voluntary and no incentives were offered. Filled questionnaires were given to the medical physicist, who returned them to the authors.

The study is exploratory in nature and descriptive statistics were generated from the data using Microsoft Excel 2007 and StatsDirect software. Descriptive statistics included summary measures and frequency tables. Collected data was handled with confidentiality.

This survey was sanctioned by the Assistant Directors of Radiography in the respective participating institutions.

## RESULTS

Sixty-three out of 205 (31%) radiographers from all the four radiology centers responded to the circulated questionnaire. Among those who responded, there were 10 student radiographers and 53 qualified radiographers employed on a full-time basis. Because of the small numbers, student radiographers participating in the survey from each hospital were combined with qualified radiographers as shown in Table 2. Possible reasons for poor response could be lack of incentive, lack of active follow-up and that radiographers working night shift were not given the questionnaire directly by the Assistant Director. Hospital D had the greatest response rate among the participating hospitals. Despite the poor response in some hospitals, the data collected provided some insights and lessons, and was, nonetheless, useful. As such, interpretations from this study should be viewed as exploratory and illuminative. Radiography techniques were not compared between the participating institutions.

**Table 2:** Response rate based on returned questionnaires according to hospitals.

Hospital	Radiographers who participated in the study			Total
	Qualified Radiographers	Student Radiographers	Total participating (%)	
A	8	3	11 (15)	73
B	21	0	21 (41)	51
C	10	1	11 (28)	39
D	14	6	20 (48)	42
Total	53	10	63 (31)	205

In terms of modalities, Hospitals A and D currently use flat panel based digital radiography units whereas Hospital B uses both computed radiography and flat panel based digital radiography units while Hospital C is currently using computed radiography units only. The equipment manufacturers are varied and included Philips Medical Systems, Siemens Medical, GE Medical Systems, Toshiba, Agfa, Fuji, Kodak and Konica Minolta. However, the interest of this present study was not to compare manufacturers.

**Figure 1:**
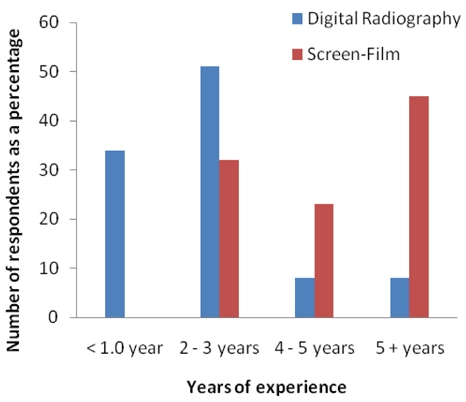
Post qualification experience stratified by imaging modality.

All the qualified radiographers had post qualification experience ranging from 1 year to more than 5 years. Post qualification experience was further stratified by whether such experience was based on using either screen-film technology or digital radiography technology. Figure 1 shows the distribution of post qualification experience according to the radiography modality. The above distribution confirms the fact that digital technology is still a relatively new technology in South African public hospitals, only 15% (8) of the qualified radiographers have 4 years or more of experience with digital radiography compared to 68% (36) for the same amount of experience with screen-film radiography.

Table 3 provides a summary of key responses from radiographers based on the questionnaire administered. This has not been broken down into qualified and student radiographers because of the small number of students involved.

**Table 3:** Summary of key responses from study by participants

Factor of interest		Number (%)	Total*
Training in digital radiography (DR)			
	Had formal education on DR	38 (61)	62
	Had formal training on DR quality control	10 (16)	61
	Thorough reading of the digital unit’s manual	14 (23)	60
	Easier to perform retakes in DR	33 (55)	60
Comparison between digital and screen-film radiography			
	Has superior spatial resolution	43 (71)	61
	Has superior image quality	45 (74)	61
	Gives relatively more radiation dose to the patient	30 (51)	59
	Has a wider dynamic range	50 (91)	55
Preference in digital radiography			
	Collimate rather than crop image	55 (89)	62
	Use grids	(100)	63

* Total – varies because some few participants did not provide responses to specific questions.

Presently radiography training in South Africa involves academic teaching at a university and clinical practice at a university hospital. Sixty-one percent (38) of the participants had been exposed to digital radiography during their lectures while at university. A small proportion, 16% (10) of the respondents underwent formal training in quality control procedures on the digital X-ray units they were using. The training was conducted by the relevant manufacturer’s representative. However, none of the surveyed departments had or were following a particular written protocol on quality control procedures, although there was a designated radiographer responsible for quality control. Twenty-three percent of the respondents had managed to read the manual of the digital X-ray unit they were operating. Slightly more than half (55%) of the participants felt it was easier for them to retake an image in digital radiography than in screen film radiography. Fifty-five percent of the respondents preferred to collimate to the region of interest instead of cropping the image after acquisition.

In an open-ended section of the questionnaire, participants were asked what they thought the advantages of digital radiography were. The responses varied, but some reported advantages were common to most participants. Table 4 shows the five popular advantages cited by the respondents. One of the commonly cited advantages of digital radiography is the increase in patient throughput. In response to the question of how many patients they could image, a median of 20 patients and 50 patients could be imaged per eight-hour shift in screen-film radiography and digital radiography with inter-quartile ranges of (15-45) and (25–108), respectively.

**Table 4:** The most commonly cited advantages of digital radiography over screen-film radiography (n=63)

Cited advantage	Number of respondents (%)
More patients treated	34 (54)
Post processing capabilities	18 (29)
Reduced radiation dose	18 (29)
Superior image quality	17 (27)
No wet processing	12 (19)
Other	34 (54)

Consistent with the fact that the participants are from teaching hospitals, the most commonly cited disadvantage of digital radiography was its ‘press button’ approach. The digital radiography user interface takes away the fundamental radiography technique training i.e. exposure settings, which is core to the art of screen-film radiography.

## DISCUSSION

This study was exploratory and illuminative for other teaching hospitals, however it has shown some very interesting results worthy of further exploration. The study also presents a potential area of collaboration with other teaching hospitals in South Africa for further studies based on lessons from this study. Although this is the case, caution is needed with the interpretations as the present sample size is relatively small given the number of radiology centers in South Africa having both conventional screen film and digital radiography. Further face-to-face interviews rather than mailed questionnaires would have improved participation. However, it stands to reason that since these surveyed institutions are teaching hospitals, their radiography practice culture cascades to a number of other centers.

Among the four institutions surveyed, only Hospital B had a picture archiving and communication system (PACS) implemented in its radiology department. Some experts have suggested that in order to reap the full benefits of digital radiography, one needs to implement PACS [12]. It is, therefore, recommended and encouraged that institutions should eventually implement PACS as they migrate from film to filmless radiography if they are to fully realize the benefits of digital radiography. Studies have shown that implementation of PACS has led to increased radiographer productivity and overall efficiency of radiology departments [13-16].

There is a large gap in the number of radiographers with at least four years of experience with digital radiography in comparison to screen-film radiography. This could be explained by the fact that most radiographers were only exposed to digital radiography after qualification. This becomes a challenge since in most cases it is the more qualified radiographers who are tasked with training students and supervising newly-qualified radiographers. Thus, it becomes imperative for them to be subjected to formal training in this modality.

Quality control procedures and quality assurance are equally important in digital radiography as they are in conventional screen-film radiography. However, it must be appreciated that the workflow process and operational nature of digital radiography directly affects traditional quality assurance practice [17]. For example, how does a radiology department implement an accurate film reject analysis in digital radiography? In South Africa, performing quality control procedures on X-ray emitting devices is enacted in law, thus it is mandatory to do such tests [18]. The Directorate: Radiation Control, which is the authority responsible for governing the use of radiation emitting substances in South Africa, has a document entitled ‘Requirements for license holders with respect to quality control tests for diagnostic X-ray imaging systems’ which lists the acceptance tests and quality control procedures [19]. It would be better if the document described how to perform these tests. It is recommended that quality control procedures required for digital radiography be included in radiography undergraduate and postgraduate programs. To further improve service delivery, radiology departments should implement formal in-house quality control training to members of staff.

Radiographers should be encouraged to read the operator’s manual of the X-ray units they are using. Reading the manual would empower the operator to realize the most out of the unit, particularly post processing functionality. Since the majority of the participants (84%) alluded to the fact that they never had formal quality control training of the units they are using, it is advisable that they at least read through the manuals available to them.

All the hospitals who participated in this study did not have a full-time medical physicist in their radiology departments. This is owing to a nationwide shortage of medical physicists in South Africa, and as a result of this critical shortage, most institutions have medical physicists working in their radiotherapy department full-time and service to diagnostic radiology departments is limited to a consultative basis. Furthermore, the regulations governing licensing and operation of radiology departments do not stipulate the minimum medical physics staffing levels consistent with the type of equipment. The advent of these new technologies should encourage participation of medical physicists who would be responsible for performing acceptance testing, patient dose measurements, objective image quality assessments, setting up of quality control programs, annual quality assessment of all the X-ray units in their departments and quality control review programs [2, 9, 10].

In dealing with radiation dose issues, it should be appreciated that digital radiography has a wider dynamic range than screen-film systems and overexposures or underexposures can yield quality images, as during post processing adjustments can be made [10]. In digital radiography, a higher patient dose would usually translate into an improvement in image quality as the images have less noise. In comparison to screen-film technology, digital radiography systems do not give an immediate feedback to radiographers concerning the radiation dose and as a result, there is a potential risk for dose creep [6, 8, 20]. Although digital radiography has the potential to achieve dose reduction in a number of examinations, patient dose increments in the range of 40–103% have been reported in the process of migrating from screen-film to digital radiography [7]. Thus, the unnecessary pursuit of beautiful images would violate the ‘as low as reasonably achievable’ (ALARA) principle. There is also a risk that if the X-ray generator automatic exposure control (AEC) develops a fault or the output calibration drifts, the dose increase or decrease can go unnoticed because of the wide exposure dynamic range of digital systems. In addition, the wide exposure dynamic range means that there is significant potential for the initial set-up of the system to be non-optimized, which further motivates for having medical physicists staffing radiology departments.

Digital radiography has post processing functions, for example, images can be cropped to show only the region of interest. It is bad radiography practice to rely on cropping images instead of collimating the beam as this leads to unnecessary radiation dose burden to the patient. In addition, proper collimating will lead to noise reduction in images, which will potentially result in lower reject rates.

## CONCLUSION

The results of this survey showed that participants are familiar with digital radiography and have embraced this relatively new technology as shown by the fact that they can identify both its advantages and disadvantages as applied to clinical practice. There is, however, minimal quality control of digital radiography being done at the surveyed institutions. It is, therefore, recommended that users of digital X-ray units adopt comprehensive national or international protocols [10, 21, 22]. Findings from this study suggest that there is need for formal education, continuing education and manufacturer training with respect to quality control as institutions make the transition from conventional screen film radiology to digital radiology. Stakeholders in the South African diagnostic radiology community should establish the minimum staffing requirements for medical physicists particularly for teaching hospitals.
